# Energy Metabolites and Indicative Significance of α-Ketoglutarate and α-Ketoglutaramate in Assessing the Progression of Chronic Hepatoencephalopathy

**DOI:** 10.3390/biom14020217

**Published:** 2024-02-12

**Authors:** Yevgeniya I. Shurubor, Andrey B. Krasnikov, Elena P. Isakova, Yulia I. Deryabina, Vladimir S. Yudin, Anton A. Keskinov, Boris F. Krasnikov

**Affiliations:** 1 Centre for Strategic Planning of FMBA of Russia, Pogodinskaya St., Bld. 10, 119121 Moscow, Russia; eshurubor@cspmz.ru (Y.I.S.); vyudin@cspfmba.ru (V.S.Y.); keskinov@cspfmba.ru (A.A.K.); 2 Independent Researcher, 24 Ivana Babushkina St., 117292 Moscow, Russia; kr-ab@yandex.ru; 3 Bach Institute of Biochemistry, Research Center of Biotechnology of the Russian Academy of Sciences, 119071 Moscow, Russia; elen_iss@mail.ru (E.P.I.); yul_der@mail.ru (Y.I.D.); 4 Department of Biochemistry and Molecular Biology, Faculty of Medicine, N.I. Pirogov Russian National Research Medical University, 1 Ostrovitianova Str., 117997 Moscow, Russia

**Keywords:** hepatoencephalopathy, thioacetamide, tricarboxylic acid cycle, ω-amidase, glutamine transaminase, α-ketoglutarate, α-ketoglutaramate, HPLC

## Abstract

In the example of a rat model with chronic hepatoencephalopathy (HE), changes in the organ morphology of rats affect the balance of metabolites of the tricarboxylic acid (TCA) cycle and metabolites of the glutamine–glutamate (Gln-Glu) cycle, namely α-ketoglutarate (αKG) and α-ketoglutaramate (αKGM), as well as the enzymes associated with them, ω-amidase (ωA) and glutamine transaminase (GTK). This model of rats was obtained as a result of 2–22 weeks of consumption by animals of hepatotoxin thioacetamide (TAA) added to drinking water at a concentration of 0.4 g/L. The control (*n* = 26) and TAA-induced (*n* = 55) groups of rats consisted of 11 cohorts each. The control cohorts consisted of 2–4 rats, and the TAA-induced cohorts consisted of 4–7 individuals. Every two weeks, samples of blood plasma, liver, kidney, and brain tissues were taken from the next cohort of rats (a total of 320 samples). By the end of the experiment, irreversible morphological changes were observed in the organs of rats: the weight of the animals was reduced up to ~45%, the weight of the kidneys up to 5%, the brain up to ~20%, and the weight of the liver increased up to ~20%. The analysis revealed: (i) a decrease in the activity of ωA and GTK in the tissues of the brain, kidneys, and liver of rats with chronic HE (by ~3, 40, and 65% and ~10, 60, and 70%, respectively); and (ii) the appearance of a significant imbalance in the content of metabolites of the Gln-Glu cycle, αKG, and αKGM. It is indicative that a ~1.5–12-fold increase in the level of αKG in the blood plasma and tissues of the organs of rats with chronic HE was accompanied by a synchronous, ~1.2–2.5-fold decrease in the level of αKGM. The data obtained indicate an essential involvement of the Gln-Glu cycle in the regulation of energy metabolism in rats under conditions of chronic HE. Attention is focused on the significance of the αKG/αKGM ratio, which can act as a potential marker for diagnosing the degree of HE development.

## 1. Introduction

Chronic hepatoencephalopathy (HE) refers to neurological disorders and occurs in patients with alcoholic or toxic liver cirrhosis [1]. This disease is characterized by mood swings, depression, decreased intellectual abilities, and reduced muscle tone [2]. There are up to five stages of development of HE, and with an III–IV degree of development of HE, the risk of death for patients increases to almost 70% [3,4].

In the development of HE, an important role is played by an increase in the level of ammonia [5,6], which, nevertheless, is only one of the triggers for the onset of this disease [7,8,9]. The liver plays a key role in maintaining the balance of ammonia in the blood, but the kidneys and brain may also be involved in its regulation. In healthy individuals, ammonia is metabolized in the liver via the urea cycle and glutamine synthetase (GS) [10]. At the same time, GS converts ammonia into glutamine (Gln), and up to ~70% of the ammonia produced by the kidneys is secreted into the renal vein, while the remaining ~30% is excreted in the urine [10]. In hyperammonemia, this ratio can be reversed [11]. The reduced ability of the liver to metabolize ammonia in hyperammonemia increases the burden on extrahepatic tissues.

The next target for ammonia exposure is the kidneys and brain [11,12]. A huge volume of blood circulates through the kidneys, but under conditions of severe HE, there is no increase in the excretion of ammonia by the kidneys, which indicates a decrease in their ability to cleanse [11]. Toxins that are not excreted by the kidneys can reach the brain through the bloodstream. As a result, in patients with HE, there may be damage to astrocytes, which normally trap and detoxify ammonia. This leads to the intracellular accumulation of Gln, the occurrence of osmotic stress, and the swelling of astrocytes [12,13]. In this case, a shift in the mitochondrial energy metabolism of cells, damage to the electron transport chain, disruption of glycolysis processes, the functioning of the tricarboxylic acid (TCA) cycle, and the balance of the glutamine–glutamate (Gln-Glu) cycle, which is involved in ammonia detoxification, can be observed [6,13,14,15,16].

A study of the brains of patients with chronic HE revealed a positive correlation between the presence of neuropsychic symptoms of the disease and the concentration of Gln formed during ammonia detoxification. At the same time, the mechanism of the development of pathology in chronic HE has not been fully elucidated. It is assumed that an increase in the level of ammonia contributes to cerebral edema and a disruption of energy metabolism in it [17,18]. However, the data obtained are difficult to interpret unambiguously. Some authors claim that ammonia directly inhibits the energy metabolism of cells, while others do not observe such a correlation [19,20,21].

Animal models are often used to study HE, monitor the disease, and test new therapeutic agents. The most common of these is the thioacetamide (TAA) model [22,23,24,25]. This model has a fairly high similarity with the development of HE in humans [22]. TAA is relatively well tolerated by animals, is less toxic, has lower mortality rates, and gives good reproducibility of results [23,24], but requires longer exposure times [26].

Standard protocols and schemes for the use of TAA in animal models have been developed in detail [25]. It is often practiced to add TAA to drinking water [27], with a duration of administration of 7 to 30 weeks [28,29,30]. Sometimes TAA is used in the form of intraperitoneal injections, suggesting better reproducibility of the results [31]. We used the method of intraperitoneal administration of TAA in our previous work devoted to the study of the restoration of the balance of basic amino acids (AA), the activity of the enzymes glutamine transaminase (GTK), ω-amidase (ωA), coenzyme A (CoA), acetyl coenzyme A (ACoA), and metabolites of the TCA cycle in rats that suffered an acute form of HE [32,33,34]. The results indicated that even after complete physiological recovery of rats, residual imbalances of AA, Gln-Glu cycle enzymes, CoA and ACoA coenzymes, as well as TCA cycle metabolites and especially α-ketoglutarate (αKG) and α-ketoglutaramate (αKGM) in the blood plasma and organs of rats were preserved. The results obtained on this rat model for studying the balance of wide-spectrum metabolites led to the idea of the possible use of metabolites of the TCA and Gln-Glu cycles for prognostic purposes to assess the severity of HE. In this regard, it was decided to test this assumption on a different rat model, namely, on a rat model with a chronic form of HE. In the presented study, we settled on the method of adding TAA to drinking water, suggesting that this approach is closer to the natural form of intake of the toxin in the body.

Thus, based on the consideration of the TAA-induced model of rats with chronic HE, the balance of TCA cycle metabolites, key metabolites of the Gln-Glu cycle, and accompanying metabolites αKG, αKGM, oxaloacetate (OA), formate (Form), pyruvate (Pyr), lactate (Lac), citrate (Cit), fumarate (Fum), and succinate (Suc), as well as the enzymes associated with them, ωA and GTK, was established. It was shown for the first time that the development of chronic HE, along with an imbalance of TCA cycle metabolites, is accompanied by dysregulation of the central enzymes of the Gln-Glu cycle, ωA and GTK, and the metabolites αKG and αKGM regulated by them, the ratio of which (αKG/αKGM) can act as an indicator of HE development.

## 2. Materials and Methods

### 2.1. Reagents

TAA and all reagents for the preparation of TCA metabolite standards, Lac and Pyr, were obtained from Sigma-Aldrich (St. Louis, MO, USA). The αKGM standard was synthesized in the laboratory, and the purity of the substance was confirmed by mass spectrometry. Ultrapure potassium phosphate dibasic powder reagent and perchloric acid (PCA, 69–72%) were obtained from J.T. Baker (Phillipsburg, NJ, USA). Ultrapure water was obtained using a Milli-Q Gradient A10 system (Millipore, Bedford, MA, USA). All chemicals were HPLC-grade and were used without further purification. For mobile phase filtration and degassing, nylon membrane filters (47 mm; pore size: 0.2 μm) obtained from Pall Life Science (Port Washington, NY, USA) were used. Reagents for enzymatic analysis: phosphate-buffered saline (PBS), paraformaldehyde, ethanol, butanol, and xylol (chemically pure) were obtained from Sigma (St. Louis, MO, USA), paraffin from Histomix (Moscow, Russia), hematoxyline from Fluka (Munich, Germany), eosin from Laborchemie Apolda GmbH (Apolda, Germany), and compound “Immuno mount” from GeneTex (Irvine, CA, USA).

### 2.2. The Model of Rats with Chronic HE

In an experiment lasting 2–22 weeks, white laboratory rats of the Wistar line, male, aged 2 months and weighing 140–150 g, were involved. Groups of control (*n* = 26) and TAA-induced rats (*n* = 55) were kept under natural light and a standard diet. Control animals received pure drinking water, and the group of TAA-induced rats consumed an aqueous solution of TAA at a concentration of 0.4 g/L. Addiction to the toxin occurred gradually, with a weekly increase in the dose of TAA in drinking water of 0.05 g/L until a final concentration of 0.4 g/L was obtained. The countdown to the experiment began on the first day of taking the final concentration of the toxin.

The physical condition of the animals was monitored on a daily basis. The first cohort of rats consisted of 2 animals in the control and 4 animals in the TAA cohorts. Starting from the 16th week, the number of control rats in the cohort was increased to 3, and in the TAA cohorts to 6–7 animals. The maintenance of the animals, the procedures performed, and the experimental protocols complied with Animal Protocols No. 22/1 approved by the Supervisory Board of the Bach Institute of Biochemistry and by the Bioethics Committee of the Biotechnology Research Center of the Russian Academy of Sciences, Moscow.

Every two weeks, rats from the next cohort were weighed, decapitated, and samples taken. Blood plasma, liver, kidney, and brain tissues were immediately frozen in liquid nitrogen and stored at −80 °C until analysis. Part of the tissue sections were sent for histology. In all samples, the specific activity of ωA, GTK, and the levels of TCA cycle metabolites were determined: OA, Form, Pyr, Lac, αKG, Cit, Fum, Suc, and αKGM.

### 2.3. Organ Histology

Histological preparations were obtained from paraffin microsections. PBS, 5× (5 tablets per 100 mL), and 4% paraformaldehyde (PFA) solution (4 g PFA per 100 mL PBS) were used for their preparation [35].

#### 2.3.1. Preparation of the Solution

To 300 mL of water (50 °C), 16 g of PFA was added, titrated with NaOH until transparent, and brought to 400 mL (pH 7.4). The mixture was filtered, poured into 50-mL Eppendorf tubes, and placed in a refrigerator.

#### 2.3.2. Preparation of Rat Organs for Embedding

Rat organs were placed for 30 min in plates with a 4% PFA solution. Then, three times, every 30 min, the PFA solution was changed, leaving the last filling for a day. Also, three times, every 30 min, the organs were washed first with a solution of PBS and then with 70% ethanol (at this stage, the organ can be left for a week at 4 °C). Then, for 40 min, the organ was washed with 80% ethanol solution, 40 min with 82% ethanol solution and 100% butanol solution (3:1), 50 min with 96% ethanol and butanol solution (1:1), 50 min with 100% butanol solution and ethanol (1:3), then twice, for 60 min, kept in 100% butanol solution.

#### 2.3.3. Embedding Rat Organs with Paraffin

Paraffin was incubated at 60 °C. The first time, the organ was embedded in paraffin for 1.5 h, then for another 5–12 h, and dried for 10–12 h.

#### 2.3.4. Preparation of Paraffin Blocks

The heated paraffin was poured into foil containers, and the organ was placed in them. After the paraffin solidified, the organ was cut out with a thin layer (1–3 mm) of paraffin around it, and with the help of heated paraffin, it was glued onto a prepared wooden block.

#### 2.3.5. Preparation of Sections for Staining

Sections of organs (25–30 μm) were made on an MS-2 sledge microtome, giving them a rectangular shape for further cutting, transferred to a glass slide, and dried for 30 min at 40 °C.

#### 2.3.6. Section Staining

Sections were paraffin-free with toluene or xylene, washed in distilled water, then in xylene (4 min) and again with fresh xylene (2.5 min), 1 min in 96% alcohol, 1 min in 70% alcohol, rinsed twice with water for 1 min, and finally washed in 0.5% hematoxylin (3 min) and 1% eosin (1 s). After staining, the compound “Immuno mount” was applied to the sections and placed under a coverslip.

### 2.4. Sample Preparation and HPLC Analysis

Frozen samples were sorted on dry ice, 20–30 mg of tissue was placed in an Eppendorf tube, weighed, and 400 µL of chilled 10% PCA was added. Brain tissues were homogenized for 6 sec at an amplitude of 15%, and liver and kidney tissues for 6 × 2 s at an amplitude of 20%. The homogenate was kept on ice for 10 min with occasional shaking, after which it was centrifuged for 20 min at 14,000× *g* (4 °C). The supernatant was centrifuged again under the same conditions; for HPLC analysis, 100–150 µL of the supernatant was added to the vial. To deproteinize blood plasma, 250 µL of chilled 10% PCA was added to 50 µL of plasma, vortexed, kept on ice for 10 min, and centrifuged as described above.

The method for determining the metabolites of TCA cycle and αKGM was described earlier [33]. Briefly, the HPLC-UV/VIS system consisted of a Waters 2489 detector, a Waters 2707 sampler with a cooled platform (4 °C), and a Waters 1525 binary pump (Waters Corporation, Milford, MA, USA). A C18 analytical column, YMC, Triart, 250 × 3.0 mm I.D., 3 µm, and a Phenomenex security guard column, C18 cartridge, 4 × 2 mm were used (Phenomenex, Torrance, CA, USA). The injected sample volume was 15 μL, the mobile phase (20 mM K_2_PO_4_, pH 2.9) was supplied in isocratic mode at a rate of 0.45 mL/min, and the wavelength of the UV/VIS detector was 210 nm. Metabolites were detected using Breeze 2 System software (Waters Corporation, Milford, MA, USA). Metabolites were quantified against external standards of known concentration.

### 2.5. Determination of GTK and ω-Amidase Activity

The measurement of enzyme activity in blood plasma and tissue samples was carried out by the spectrophotometry method developed for a 96-well plate.

#### 2.5.1. GTK Endpoint Assay

The reaction mixture (RM) for the assay contained 50 mM ammediol (Amm), 2.5 mM α-keto-γ-methiobutyrate (KMB), 10 mM L-phenylalanine (Phe), and a sample of 5 µL. The total RM volume was adjusted with H_2_O to 50 µL. The blank contained no Phe (H_2_O instead). Samples were incubated at 37 °C for 1 h. The reaction was stopped by the addition of 150 µL of 1 M NaOH. Absorbance was read within five minutes at 322 nm (due to phenylpyruvate enol ε_322nm_ = 16,000 M^−1^ cm^−1^). Spectrophotometric determinations were conducted with a SpectraMax 96-well plate spectrophotometer (Molecular Devices, Sunnyvale, CA, USA).

#### 2.5.2. ω-Amidase Endpoint Assay

The RM contained 5 mM αKGM, 5 mM DTT, 100 mM Tris-HCl buffer (pH 8.5), and an enzyme source. The final RM volume was adjusted with H_2_O to 50 µL. Note that when the assays were conducted with purified ωA, the blank contained complete RM lacking enzyme. For assays of crude tissue/cell homogenates, the blank contained the complete RM plus homogenate and 200 mM glycylglycine. After 5–30 min incubation at 37 °C, the reaction was terminated by the addition of 20 µL of 5 mM 2,4-dinitrophenylhydrazine in 2 M HCl. After a further incubation for 5 min at 37 °C, 130 µL of 1 M NaOH was added, and the absorbance was read at 430 nm within 5 min. The ε_430nm_ of α-ketoglutarate*2,4-dinitrophenylhydrazone under these conditions was 16,000 M^−1^·cm^−1^.

### 2.6. Statistical Analysis

For statistical analysis, 13 types of measurements (concentrations of metabolites, specific enzyme activities, and the αKG/αKGM ratio) in 4 types of samples (blood plasma, liver, kidney, and brain tissue) for each rat were used as input data. The rats were represented by 11 cohorts, where each subsequent cohort was 2 weeks older than the previous one. All rats were divided into 2 groups: TAA-induced and control. The number of rats within one cohort varied from 2–3 in the control group to 4–7 in the group of TAA-induced rats. To prevent bias in the results, if data on a combination of sample type and measurement type were missing in at least one of the cohorts, then data for all cohorts and the corresponding combination of sample type and measurement type were removed from the analysis. Thus, a total of 2995 raw measurements were obtained.

The analysis took into account that with age, animals undergo a natural change in their metabolic profile, which is determined by the duration of the experiment (from 2 to 22 weeks). That is, age-related variability is added to the initial individual variability of animals.

To begin with, using the Shapiro–Wilk test (taking into account the condition of having more than 3 examples in a group), hypotheses were tested for the normality of the distribution of data obtained during the experiment for each combination: group type, sample type, and measurement type. With a large number of such combinations, it is necessary to test many hypotheses. A total of 74 such hypotheses were tested, of which 43 were rejected at a significance level of 5%. The Benjamini–Hochberg procedure was used to control for the False Discovery Rate (FDR) error in multiple hypothesis testing. However, when adding the time parameter (cohort), the number of hypotheses increased to 399, and none of them were rejected with an FDR of 5% significance level.

Next, using the Bartlett test (taking into account the condition of having at least 2 examples in the group), hypotheses were tested for equality of variances in the TAA-induced and control groups of animals for the same combinations (type of measurement, type of sample, and cohort). A total of 407 such hypotheses were tested, of which five were rejected with FDR control at a significance level of 5%. These five positives represent about 1% of the total number of hypotheses and may well be false; however, the approach chosen below will not rely on the property of equality of variances as necessary.

Note: The total number of hypotheses in the tests for normality and equality of variances was different because if combinations of measurement type, sample type, and cohort did not have the minimum number of examples to apply the test, then that combination was excluded from testing.

Thus, it was concluded that:

1. Based on the results of the Shapiro–Wilk and Bartlett tests in each individual cohort, the data obtained for the types of measurements and types of samples for TAA-induced animals are the result of a shift in the normal distribution of values while maintaining the original variance of their corresponding control groups of animals. This is true for ~98.8% of the considered about four hundred combinations of cohorts, types of measurements, and types of samples.

2. Variance values may differ between cohorts. This was tested using the Bartlett test by removing the cohort factor from each combination of group type, sample type, and measurement type. According to the results of such testing, 23 out of 74 hypotheses were rejected.

3. The analyzed data are two matched cohorts; that is, the test and control can only be compared within the same cohorts.

4. The parametric method of comparing the means of two normal samples of different sizes with an unknown equal variance cannot be applied to all cohorts. This limitation is a consequence of insufficient data, as the number of animals in both TAA-induced and control groups varied between cohorts.

5. Animals within the TAA-induced and control groups within the same cohort are independent and can be compared to each other.

6. With rare exceptions, the differences in values between random animals from the test and control groups represent a normal distribution within any selected cohort (this is a natural consequence of the normality of the distributions in each of the groups), and as a consequence, the median coincides with the mean value of the difference. However, in the aggregate, when all cohorts are combined into one sample, the distribution of the difference in values is not normal. This statement was true for 30 of the 37 combinations of measurement types and sample types, which was also verified using the Shapiro–Wilk test.

Taking into account the limitations outlined in paragraph 4 and the opportunities provided by paragraphs 5 and 6, an approach was then taken to identify possible differences between the test and control groups of animals using non-parametric tests for pairwise comparisons between animals of different groups within the same cohort.

At the first stage, using the sign test, the hypothesis that the median of the difference in measurements in the groups of TAA-induced and control rats is equal to zero was tested. If for an arbitrary cohort we introduce the designations nC for the number of measurements in the control and nT for the number of measurements in the group of TAA-induced rats, then for this cohort we obtain the number of different differences between the values from the test group and the control equal to nC × nT. Thanks to this approach, we obtain several times more observations than the group sizes, thereby reducing the dispersion of the resulting estimates. In addition, relying on such a property of the test as nonparametricity, we are able to combine together samples of pairwise differences collected within cohorts. Thanks to this, we can test the hypothesis that, within a randomly selected cohort, the probability of observing a negative difference does not differ significantly from the probability of observing a positive difference. Or in the formal notation H_0_: Median [T − C] = 0 ↔ P(T > C) = ½ (and a symmetric version of the null hypothesis formulation P(T < C) = ½). For the combinations of measurement types and tissue types considered, we obtained 37 hypotheses about the equal probability of the sign of the difference between the TAA-induced and control groups in a random cohort. Of these, 28 hypotheses were rejected, which allowed us at this stage to select those combinations of measurement types and sample types for which we further obtained quantitative estimates of the deviations of the test group from the control group. Table 1 shows the data (AVG ± STD) and confidence intervals at the 5% significance level for P(T < C), that is, the probability that when selecting a random pair of animals from the TAA-induced and control groups within an arbitrary cohort, a measurement from the test group was lower than in the control. The values in Table 1 are presented only for those cells where the hypothesis H_0_: P(T < C) = ½ was rejected with FDR control at the 5% significance level.

## 3. Results

### 3.1. The Pathophysiology, Morphology, and Histology of the Organs of Rats

**Figure 1:**
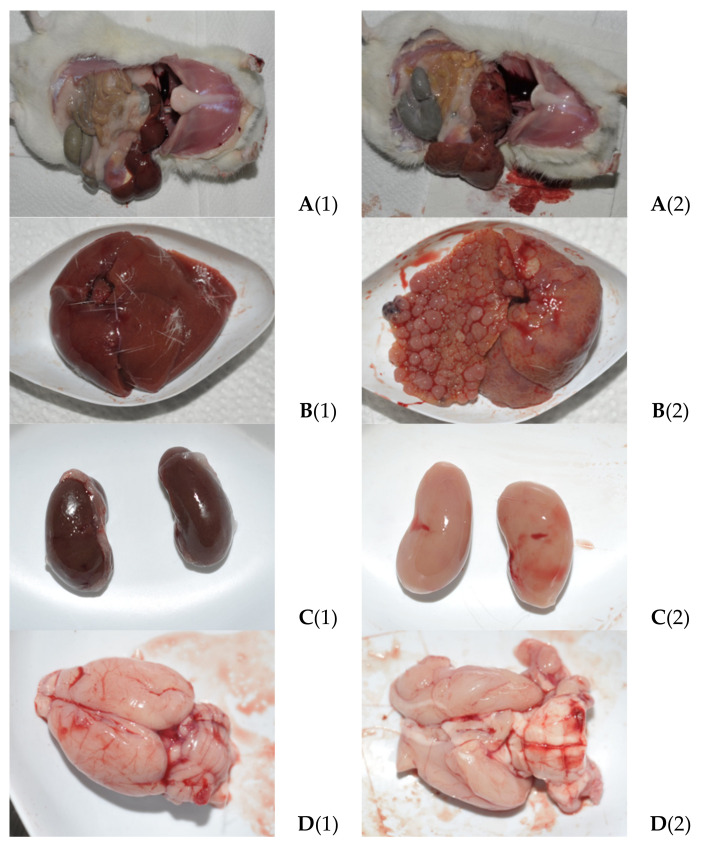
The appearance of the abdominal cavity (**A**), liver (**B**), kidneys (**C**), and brain (**D**) of the control (1) and TAA-induced group (2) animals at the 20th week of the experiment.

Two weeks after the start of administration of an aqueous solution of TAA, the behavior of the experimental rats visually remained the same, although there were slight physiological changes and a lag in weight gain. After 4 weeks, the weight of the experimental rats was reduced to 185–188 g against 275–302 g in the control group. The liver became paler, the kidneys acquired a pale brown color, the intestines were slightly swollen, and fatty tissue was almost absent. After 6 weeks, the behavior of the animals also remained almost unchanged, but weight loss and increased signs of pathophysiology continued. The liver became lighter, with yellowish inclusions and signs of structural fibrosis. The kidneys also became lighter, with a greenish-brown tint. The stomach and small intestines remained awake, and the animals seemed to have somewhat adapted to the effects of TAA. By the 8th week, the weight of the rats was slightly more than 70% of the weight of the animals in the control group. Small hematomas appeared in the liver; the color became serous with yellowness; the surface was rough; but the organs of the digestive system still remained without noticeable changes. By the 10th week, the weight of the animals continued to decrease. Fragments of fibrosis, hematomas, and fatty inclusions appeared in the liver. The kidneys became pale yellow with a greenish tint and looked slightly larger than the controls. The brain was softened, but there were no visible changes in the digestive organs. By the 12th week of the experiment, the body weight of rats had decreased to ~60% of the mass of control animals. In the liver of rats, signs of necrosis, the presence of cirrhotic areas, an increase in hematomas, and fatty inclusions appeared. The kidneys, brain, and digestive organs did not visually differ from the previous groups, with the exception of the small intestine, in which minor changes were noted, indicating the appearance of an inflammatory process. After 14 weeks in the liver of rats, the number of areas with cirrhosis, the presence of hematomas, and adipose tissue increased. The liver of some animals was hypertrophied, the structure was changed, and cellularity appeared. The color of the kidneys changed to pale yellow or greenish, and edema and cysts of unknown etiology appeared. The revealed structural changes in the organs of rats indicate an appearance by the 20th week of the experiment of stable and irreversible signs of the development of chronic HE (Figure 1).

Mortality in the group of experimental rats during the observation period was ~2%; hair loss was observed in ~10% of animals. The weight of the experimental animals by 16–20 weeks was stabilized and even slightly increased relative to the previous indicators, which is probably due to the relative maturation of the rats. However, weight loss by 16 and 20 weeks was up to ~35–45%. Kidney weight was reduced by ~5–6%, brain weight was reduced by ~5–20%, and liver weight was increased by ~10–20% (Table 2).

**Figure 2:**
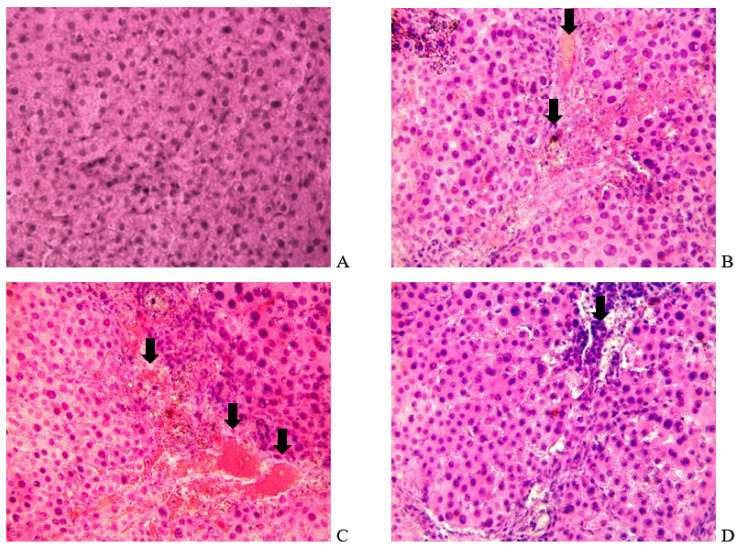
Liver sections of control (**A**) and TAA-induced (**B**–**D**) rats 4 weeks after the initiation of the TAA aqueous solution. Panel (**B**): small droplet fatty degeneration is indicated by arrows. Panel (**C**): the arrows indicate diapedetic hemorrhages. Panel (**D**): the arrow indicates the foci of small-cell formations. The tissues were stained with hematoxylin–eosin; magnification: ×400.

As part of monitoring the development of HE, tissue sections of animals were examined in the 4th week of the experiment. On sections of the livers of the control animals (Figure 2A), hepatocytes had normal cytomorphology, well-defined nuclei, and moderate tissue polymorphism. Sections of the liver of experimental rats showed signs of small-drop fatty degeneration (Figure 2B) and diapedetic hemorrhages (Figure 2C), and foci of small-cell formations indicating the presence of inflammatory processes were visible (Figure 2D). A small part of hepatocytes (~10–12%) acquired apoptotic signs. The data obtained correspond to the symptoms of liver pathology after exposure to TAA and carbon tetrachloride (CCl_4_) [36].

**Figure 3:**
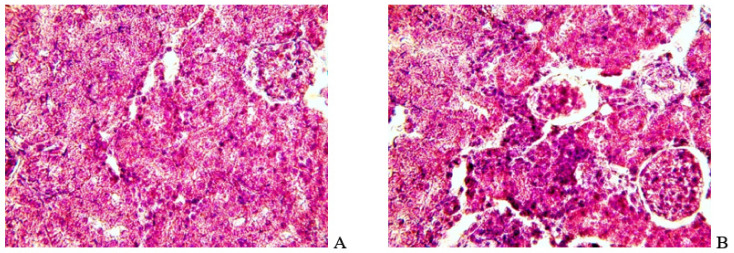
Kidney tissue sections of control (**A**) and TAA-induced (**B**) rats after 4 weeks of taking an aqueous solution of TAA. Tissues were stained with hematoxylin–eosin; ×400 magnification.

Sections of kidney tissues of rats with chronic HE (Figure 3A,B) had looser tissues of the pelvis (internal part of the organ) than in the control, impaired glomerular structure, and the appearance of microhemorrhages. In the tissues of the parenchyma, densification of cellular structures and the formation of furnaces, which are not characteristic of healthy tissue, were noted. When the organ is opened, its swelling is noticeable.

**Figure 4:**
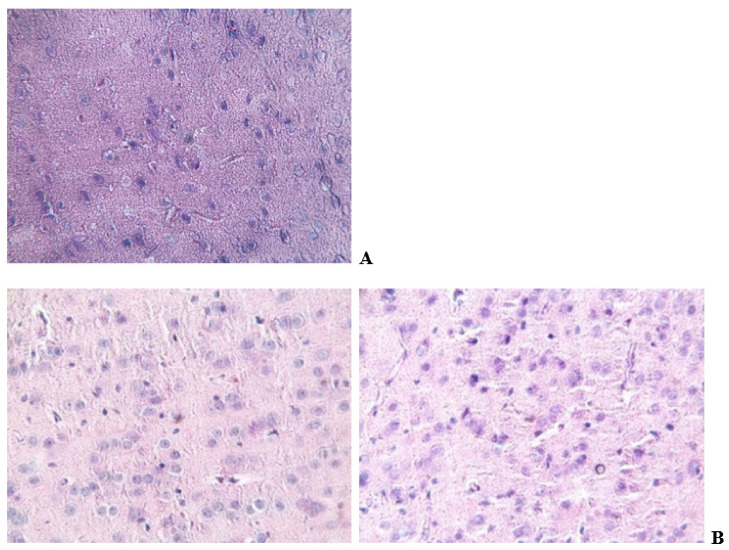
Brain tissue sections of control (**A**) and TAA-induced (**B**) rats after 4 weeks of taking an aqueous solution of TAA. Tissues were stained with hematoxylin–eosin; ×400 magnification.

In sections of the brain tissues of experimental rats (Figure 4A,B), loosening and formation of intercellular cavities (hydrops of the brain) with the appearance of foci of inflammation and hematomas were noted.

### 3.2. Measurements Taken

In samples of blood plasma, tissues of the liver, kidneys, and brain of control and TAA-induced rats, the activity of ωA and GTK, the levels of TCA cycle metabolites, and associated keto acids, namely: OA, Form, Pyr, Lac, Cit, αKG, αKGM, Fum, and Suc, were determined. For better visualization of the obtained results, the data were presented as the AVG ± STD for the groups (control and TAA-induced) and cohorts (2–22 weeks; 11 cohorts in total) of rats.

#### 3.2.1. ωA and GTK Activity

**Figure 5:**
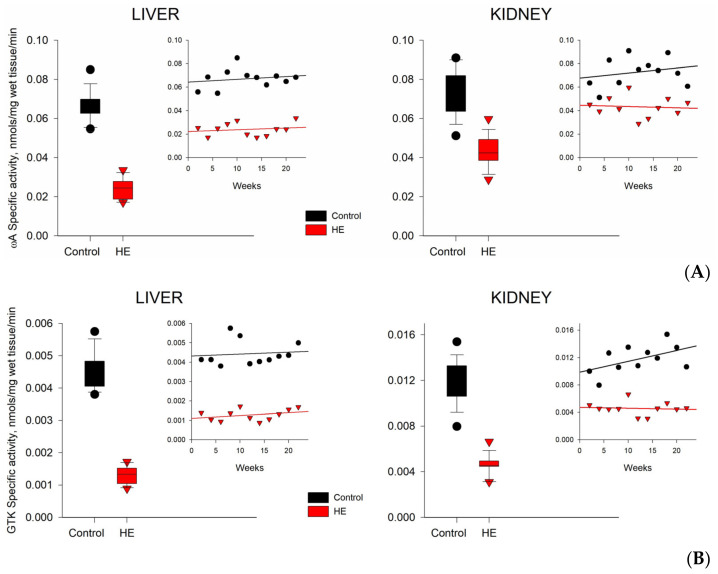
Specific activity (nmols/mg of wet tissue/min) of ωA (**A**) and GTK (**B**) in liver and kidney tissues of control (black circles) and TAA-induced (red triangles) rats. The data are presented as average values (for the period of 2–22 weeks).

The specific activity of ωA and GTK plays an important role in the regulation of cellular homeostasis and the neutralization of ammonia during the development of chronic HE. In the blood plasma and tissues of the control groups of rats, the level of ωA activity was noticeably higher than the level of GTK activity, which was already noted earlier [34]. The highest level of enzyme activity was noted in the tissues of the kidneys and liver, and the lowest in the tissues of the brain and blood plasma of rats (Table 3). With the development of chronic HE, the activity of enzymes in the tissues of the liver and kidneys of rats significantly decreased, while in the tissues of the brain and blood plasma, the changes were minimal (Table 3; Figure 5A,B and Figure S1).

In the blood plasma of the control groups of rats, the ωA activity was ≥five times higher than the GTK activity (Table 3 and Figure 5A,B). In groups of rats with chronic HE, this difference somewhat decreased due to the different sensitivity of enzymes to the effects of TAA. In the liver tissues of the control groups of rats, the specific activity of ωA was ≥15 times higher than the activity of GTK. In rats with chronic HE, this difference increased up to ≥18 times, which was due to a greater inhibition of GTK activity relative to ωA activity. In the kidney tissues of the control groups of rats, the ωA activity was ≥six times higher than the GTK activity, and in the groups with chronic HE, it was ≥nine times higher, which was also due to a large decrease in the GTK activity relative to the ωA activity. In the brain tissues of the control groups of rats, the specific activity of ωA was ≥two times higher than the activity of GTK. Under the conditions of the development of chronic HE, the activity of both enzymes in the brain tissues and in the blood plasma of rats changed to a much lesser extent than in the tissues of the liver or kidneys (Figure 5 and Figure S1).

#### 3.2.2. TCA Cycle Metabolites

The highest total and average values of the pool of TCA metabolites in the control and TAA-induced groups of rats were characteristic of brain tissues, where they were ≥2.5 times higher than the values in liver and kidney tissues (Table 4). Moreover, with the development of chronic HE, in the brain tissues and in the blood plasma of rats, there was a slight decrease in the pool of TCA cycle metabolites (~7% and ~16%, respectively), while in the tissues of the kidneys and liver, a slight increase was observed (~7% and ~12%, respectively).

**Figure 6:**
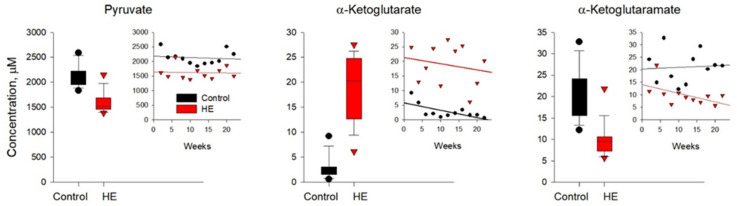
Plasma levels of pyruvate, α-ketoglutarate, and α-ketoglutaramate in the control (black circles) and TAA-induced groups of rats (red triangles). Data are presented for the statistically significant metabolites as average values over a period of 2–22 weeks.

In the blood plasma of rats with chronic HE (Figure 6 and Figure S2), the levels of the Pyr, Lac, OA, and Fum pools were ~15–25% lower than the control values. The maximum decrease (~two times) was observed in αKGM. At the same time, the levels of such metabolites as Cit, Mal, and Form increased by about the same amount. The most impressive increase in plasma levels of αKG (up to ~7-fold) in TAA-induced rats was observed. Due to the high biological variability of metabolites, the average values of which were calculated over the period from 2 to 22 weeks, a significant difference between the control and experimental groups of animals was found only for Pyr, αKG, and αKGM (*p* < 0.05), as shown in Figure 6.

**Figure 7:**
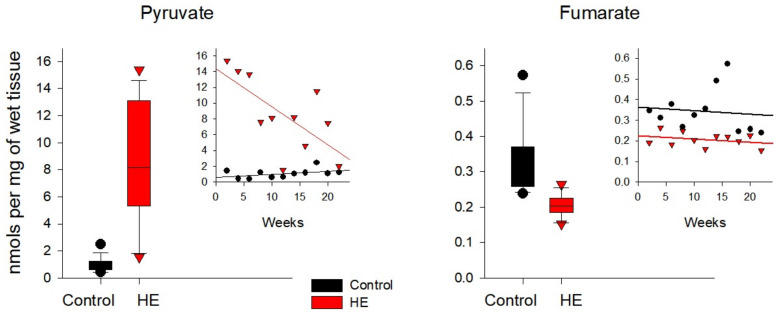
Levels of pyruvate and fumarate in the liver tissues of the control (black circles) and TAA-induced groups of rats (red circles). Data are presented for the statistically significant metabolites as average values over a period of 2–22 weeks.

In the livers of rats with chronic HE (Figure 7 and Figure S3), the levels of Lac, Fum, and αKGM were reduced by ~15–40% of control values. At the same time, in the same groups of rats, the concentrations of such metabolites as Pyr, OA, Cit, and αKG were ~4–12 times higher than the control values. Curiously, the concentration of αKG in the liver of control rats was minimal. In the liver of rats with chronic HE, the absolute concentration of αKG was also low, but almost an order of magnitude higher than the control values.

**Figure 8:**
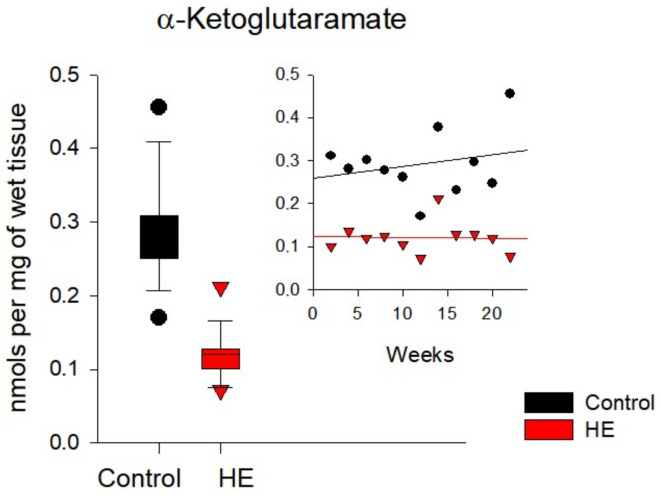
Levels of α-ketoglutaramate in the kidney tissues of the control (black circles) and TAA-induced groups of rats (red triangles). Data are presented for the statistically significant metabolites as average values over a period of 2–22 weeks.

In the kidneys of rats with chronic HE (Figure 8 and Figure S4), there was a ~20–60% decrease in the concentrations of Pyr, OA, and αKGM. Conversely, the concentrations of Lac, Cit, αKG, and Suc exceeded the control values by ~10–80%. The maximum multidirectional changes were typical for αKGM and αKG (~60 and ~80%, respectively).

**Figure 9:**
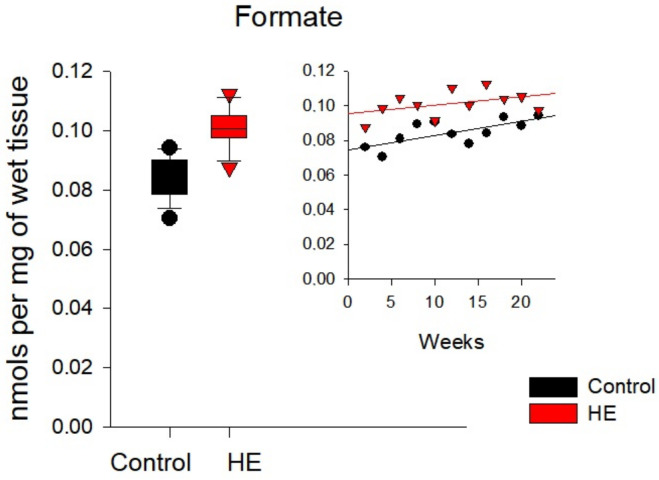
Formate levels in the brain tissues of the control (black circles) and TAA-induced groups of rats (red triangles). Data are presented for the statistically significant metabolites as mean values over a period of 2–22 weeks.

In the brain tissues of the control and TAA-induced groups of rats (Figure 9 and Figure S5), the average concentrations of metabolites differed not so significantly as in the liver and kidneys. The concentrations of Pyr, Lac, OA, Fum, and αKGM in the brain tissues of rats with chronic HE decreased by ~5–60% relative to control values. The concentrations of Form, Cit, and αKG, on the contrary, increased by ~25–35% relative to the control. The maximum decrease was noted for OA (~60%) and the maximum increase for αKG (~35%).

### 3.3. Biological Variability of the ωA, GTK, and TCA Metabolites

Biological variability of ωA and GTK (CV, %) in plasma, kidney, liver, and brain tissues of control rat cohorts ranged from ~1 to 100%. With the development of chronic HE, an increase in CV was observed, which, apparently, is associated with different individual responses of animals to the toxin. At the same time, if the CV of the specific activity of GTK in the brain tissues and in the blood plasma of TAA-induced rats increased by only ~30%, then in the tissues of the kidneys and liver it already exceeded the control values by ~2–4 times. The CV of the specific activity of ωA in the brain tissues of rats with chronic HE also increased slightly (up to ~30%), but in the tissues of the kidneys, liver, and blood plasma it increased by the same ~2–4 times.

The biological variability of individual TCA metabolites in the plasma, kidney, liver, and brain tissues of rats from the control cohorts was ~10–130%. The highest levels of CV were noted in the tissues of the liver and kidneys of rats, and the lowest were in the tissues of the brain. With the development of chronic HE, the variability of TCA cycle metabolites in the liver and kidney tissues of rats increased by ~14–16%, and in blood plasma and brain tissues by ~25–45%. It should be noted that the biological variability of metabolites in rat blood plasma was comparable to the variability we obtained earlier for human blood plasma [37]. Thus, it can be assumed that the specific activity of ωA and GTK is more vulnerable (higher CV) to the effects of TAA than bioenergetic metabolites, in particular, αKG and αKGM, partially controlled by these enzymes.

### 3.4. Statistical Analysis

The principles of statistical data processing and the details of the statistical justification are set out in Section 2. In Table 5, we are given the mathematical expectations of deviations in the levels of metabolites in samples of rats from TAA groups from similar levels in samples of rats from control groups (value deviation ∆ = E(m((T − C)/C)), where E - is the mathematical expectation, m - is the cohort median, T - is the value for an individual from the test group, and C - is the value for an individual from the control group. The fraction (T − C)/C is conveniently interpreted as the deviation of the test value from the control value, expressed as a fraction of the control value (or as a % when multiplying this value by 100%). Estimates of deviations are given only for those types of samples and types of measurements for which the probability P(T < C) ≠ ½ when selecting a random pair of animals from the TAA-induced and control groups (see Table 1 in Section 2.6).

Table 5 shows data in the format (AVG ± STD) and interpretation of deviation scale.

Calculations performed to assess the mathematical expectations of deviations in the levels of specific activity of enzymes ωA and GTA in samples of TAA-induced rats from similar levels in samples of control rats (%) showed an almost two-fold statistically significant decrease in the specific activity of enzymes in the tissues of the liver and kidneys of rats with chronic HE (Figure 5 and Table 5). At the same time, similar calculations performed for blood plasma and brain tissue of rats with chronic HE did not reveal statistically significant differences from the control (Figures S2 and S5; also see Table 1 in Section 2.6).

Calculations to assess the mathematical expectations of deviations in the levels of metabolites in the blood plasma of rats with chronic HE from the control ones revealed a decrease in the level of Pyr by a quarter and an increase in the level of αKG by an order of magnitude at a significance level of 5%, as well as a change in the level of Cit (the fact of change is at a significance level of 5%, and the fact of growth by 14% is at the significance level of 13%). The mathematical approach used permitted us to add Cit to the list of previously identified metabolites with a statistically significant difference of 5% between the groups of TAA-induced and control animals (Figure 6 and Table 5).

Similar calculations performed for rat liver tissues made it possible to determine an almost two-fold decrease in the level of Fum and an increase by an order of magnitude in the level of Pyr at a significance level of 5% (Figure 7), as well as an increase by an order of magnitude in the level of Cit in the liver of rats with the development of the disease (Table 5). In the kidney tissues of TAA-induced rats, an almost two-fold decrease in the level of αKGM was confirmed with the development of chronic HE (Figure 8 and Table 5), and in the brain tissues with the development of the disease, an increase in the level of Form by almost 20% was confirmed (Figure 9 and Table 5).

## 4. Discussion

A long-term use of TAA leads to the development of chronic HE, changes the metabolic balance of the cell, causes oxidative stress, inhibits the mitochondrial respiratory chain, and depresses the central nervous system [14]. There is an assumption that in chronic liver disease, not only ammonia stimulates the development of HE but also other metabolites associated with the development of this disease [38]. For example, an increase in the levels of excitotoxic Gln, Glu, and ROS products in brain tissues can also affect the deterioration of the central nervous system [14,38]. With the development of HE, the concentration of Gln can significantly increase in brain tissues, change the permeability of mitochondria in astrocytes, and contribute to the development of mitochondrial dysfunction [39,40]. Here, in addition to a direct effect on the Gln-Glu cycle, inhibition of ωA and GTK, and a toxic effect on the work of the TCA cycle, inhibition of such enzymes as pyruvate dehydrogenase (PDH) and α-ketoglutarate dehydrogenase (αKGDH) was noted with simultaneous stimulation of phosphofructokinase and other glycolytic enzymes of brain tissue [34,41].

In this regard, we can focus on the experiment with the use of Cit (a direct precursor of αKG) in healthy volunteers and patients with liver cirrhosis. In the intestines of healthy volunteers, Cit was transformed into αKG and was rapidly eliminated from the body, and in patients with chronic liver disease, the accumulation of αKG in the blood was observed [42]. Apparently, an excess of ammonium slowed down the rate of TCA cycle work through the inhibition of αKGDH, which ensures the availability of αKG for ammonia binding and the formation of Gln [5].

A similar trend of increasing the level of αKG in the blood plasma and tissues of rats with chronic HE was also noted in our experiment. Apparently, this is due not only to the inhibition of αKGDH in rats with chronic HE. According to the data we obtained (Table 3), during the development of chronic HE in rats, there is an inhibition of two other enzymes that regulate the work of the Gln-Glu cycle and control the levels of formation of αKG and αKGM, namely: ωA and GTK. It is noted that the inhibition of αKGDH and αKG can be overcome by the use of specific additives necessary to maintain the rest of the TCA cycle. Branched-chain amino acids (e.g., isoleucine) may be such supplements, which are often recommended for patients with HE [43].

In addition to αKGDH, ammonia can inhibit PDH, reducing its availability for the normal functioning of the TCA cycle and thus affecting the cerebral transport of glucose and creatine [44]. In animals with developed HE, the deactivation of oxidative glucose metabolism and activation of cerebral anaerobic glycolysis, which reduces the rate of glucose passage through oxidative phosphorylation, are often observed [44]. Such a change in energy metabolism in brain cells can lead to an increase in Lac production even in the presence of sufficient oxygen [44].

Monitoring of pathophysiological changes in the vital organs of animals during the development of chronic HE indicates the appearance in the second half of the experiment of stable and irreversible signs of necrotic transformations. The identified morphological transformations were accompanied by changes in the activity of GTK and ωA, as well as in the balance of energy metabolites in the TCA cycle.

Pyr is an important metabolite that combines several metabolic pathways at once. This metabolite can be converted to carbohydrates via gluconeogenesis, to fatty acids or energy via acetyl-CoA, or to the amino acid alanine or ethanol. In the brain tissues of patients with HE, an increase in the level of Lac is also possible, and whether this increase is a cause or a consequence of the development of HE is not yet clear [45]. An increase in the level of Lac is possible as a result of blood intake, increased glycolysis, increased production, and/or decreased utilization of the metabolite itself [45]. Other authors noted an increase in the activity of the pyruvate dehydrogenase complex (PDHC) and lactate dehydrogenase (LDH), which was accompanied by an increase in the concentrations of acetyl-CoA and Lac. Inhibition of PDHC and LDH led to a decrease in liver damage and, accordingly, a decrease in mortality in mice [46].

The analysis of the levels and distribution of Pyr and Lac in the blood plasma and organs of rats with chronic HE indicates their different metabolic fates depending on the localization. Thus, with the development of HE, the levels of Pyr and Lac in the blood plasma and brain tissues of rats synchronously decreased, while in the liver and kidneys of rats, they changed in different directions. An increase in the level of Pyr in the liver tissues was accompanied by a decrease in the level of Lac, and vice versa, a decrease in the level of Pyr in the tissues of the kidneys was accompanied by an increase in the level of Lac.

At the same time, the trends in the distribution of Pyr in the blood plasma and tissues of rats with chronic HE repeated the trends in the distribution of OA. The synchronous increase or decrease in the concentrations of Pyr and OA was most likely due to their close metabolic relationship: Pyr is an important substrate for OA, which, as a result of a series of anaplerotic reactions, replenishes the TCA cycle with intermediate metabolites.

Another metabolite directly associated with Pyr is Form, whose levels of formation are regulated by microbiome metabolism, type of nutrition, the presence of toxicological effects, etc. [47]. The role of Form has not yet been fully assessed, but it is known that serine, glycine, and formaldehyde, which is formed from methanol, are its precursors. Form also induces metabolic switching in purine, pyrimidine, and energy metabolism, and its physiological level in animal blood plasma varies over a wide range (10–100 µM) [47,48]. With an increase in the rate of glycolysis and an increase in the level of Lac, a Form-dependent increase in ATP levels can be observed [48].

In the blood plasma and brain tissues of rats with chronic HE, the pattern of the distribution of Form was opposite to that of Pyr, Lac, and OA. Meanwhile, in the liver and kidneys of rats, the trends of its distribution coincided with the distribution of Pyr and OA, but not Lac. In general, in the blood plasma of rats with chronic HE, a decrease in the levels of Pyr, Lac, OA, αKGM, and Fum was noted, along with a slight increase in the level of Form and a noticeable increase in the levels of Cit and αKG.

In the liver tissues of rats with chronic HE, a significant increase in the levels of Pyr was noted against the background of a slight increase in the level of Form and a slight decrease in the level of Lac. It is noteworthy that the increase in the level of Pyr was accompanied by an increase in the level of OA and a decrease in the level of Fum. The levels of Cit and αKG in the liver of rats with chronic HE, as well as in blood plasma, significantly increased against the background of a slight decrease in the level of αKGM.

In the kidneys of rats with chronic HE, a slight (~20%) decrease in the level of Pyr and Form was accompanied by a slight (~10%) increase in the level of Lac, probably due to the partial inhibition of PDH. The low levels of formation of Pyr were also manifested in the low levels of formation of OA and Fum, which indicates some inhibition in the functioning of this part of the TCA cycle. At the same time, the metabolites of another part of the TCA cycle (Cit, αKG, and Suc) in the kidneys of rats with chronic HE gravitated towards a certain accumulation. It can be assumed that this disturbance in the functioning of the TCA cycle may be the result of the partial inhibition of αKGDH under the influence of excess ammonia, which affects the utilization of Cit and reduces the availability of αKG for the formation of Gln [5]. In addition to αKGDH inhibition, ammonia affects the activity of ωA and GTK, the main enzymes of the Gln-Glu cycle, and, as noted above, under conditions of chronic HE, GTK was inhibited to a greater extent than ωA. GTK regulates the conversion of Gln into αKGM, while ωA is responsible for the transformation of αKGM to αKG. It is interesting to note that with an increase in the level of Cit in the kidneys of rats with chronic HE of ~20%, the concentration of αKG almost doubled. It can be assumed that the accumulation of αKG was due not only to the inhibition of αKGDH but also to the inhibition of the enzymes of the Gln-Glu cycle. A clear shift in the balance in this cycle is evidenced by an increase in αKG levels with a synchronous decrease in αKGM levels.

A slight decrease in the levels of Pyr, Lac, OA, Fum, and αKGM in the brain tissues of rats with chronic HE was accompanied by a slight increase in the level of Form. At the same time, the level of Cit and αKG in the brain tissues of rats was also higher than the control values with a stable content of Suc. The accumulation trends of these TCA cycle metabolites in the brain tissues and in the blood plasma of rats were similar but differed from those in the tissues of the kidneys and liver. The only exceptions were such metabolites as αKG and αKGM, the trends of which were similar in all samples of rats with chronic HE without exception, namely: αKG levels significantly increased against the background of a synchronous decrease in αKGM levels.

Briefly, the mechanism of conversion of αKGM and αKG with the participation of the enzymes ωA and GTK is as follows: the production of αKG from Gln (glutaminase II pathway) occurs by transamination of Gln into the intermediate product αKGM, which occurs with the participation of GTK. Then, with the participation of the ωA enzyme, αKGM is hydrolyzed to αKG and ammonia [49].

In general, the monitoring of the content of the intermediate metabolite αKGM deserves closer attention. An increase in the level of αKGM in the urine of patients with congenital disorders of the urea cycle has already been noted [49]. The authors made an assumption about the possibility of using this metabolite as a biomarker for diagnosing diseases associated with hyperammonemia and urea cycle disorders [50].

The distribution trends of αKG and αKGM in the blood plasma and tissues of rats with chronic HE are closely related to the activity of the enzymes regulating their levels. As shown in Table 3, the specific activities of ωA and GTK in the tissues of the kidneys and liver of rats during the development of chronic HE decreased by ~2 and 3 for ωA, and by ~3 and 4 times for GTK, respectively. Differences in the decrease in the expression of these enzymes may indirectly indicate a lower sensitivity of the rat kidneys to the effects of the toxin compared to the liver. In the blood plasma and brain tissues of rats with chronic HE, changes in the activity of GTK and ωA were not so significant (Figure 5). The specific activity of GTK turned out to be more vulnerable to the effects of the toxin than ωA, which may be due to its initially lower activity in tissues. Thus, in control samples of the brain tissue and blood plasma of rats, the activity of GTK was lower than the levels of ωA activity by ~2.5 and ~5 times, and in the kidney and liver tissues of rats by ~6 and ~15 times, respectively. With the development of HE, this discrepancy only increased.

Trends in the accumulation of key metabolites of the Gln-Glu cycle, such as αKGM and αKG, regulated by the activity of GTK and ωA, also changed with the development of chronic HE. If the concentration of αKGM in the blood plasma of rats with chronic HE decreased by ~2.5 times relative to the control values, then the level of αKG increased by ~7 times. As a result, the ratio of both metabolites (αKG/αKGM) in blood plasma, reflecting the discrepancy between their values during the development of pathology, increased from 0.13 in the control group to 1.97 in the group of rats with chronic HE, that is, more than 15 times. At the same time, the difference in the levels of specific activity of both enzymes in blood plasma was small. Obviously, it can be assumed that such a significant change in the levels of αKGM and αKG in blood plasma is due not so much to the activity of the GTK and ωA enzymes as to the redistribution of metabolites from other organs.

The concentrations of αKGM and αKG in the brain tissues of rats with chronic HE, in contrast to blood plasma, changed insignificantly. Thus, the concentration of αKG increased here by ~1.4 times relative to the control, while the concentration of αKGM, on the contrary, decreased by ~1.3 times. In this regard, the αKG/αKGM ratio changed by two times, from 0.016 in the control to 0.032 in the group of rats with liver pathology. The activity of ωA and GTK in the brain tissue of rats with chronic HE also decreased slightly, by ~3–10%, which was lower than the levels of change in αKG and αKGM.

In the liver of rats with chronic HE, the decrease in the specific activity of ωA and GTK, compared with the control, was maximum and reached ~65% and ~70%, respectively. At the same time, the ωA activity was ~15–18 times higher than the GTK activity both in the control group and in the group of rats with chronic HE. The level of αKGM regulated by GTK in the group of rats with liver pathology was slightly (~1.2 times) lower than the control values. Perhaps this was due to the higher activity of ωA, which regulates the conversion of αKGM to αKG. In contrast to a slight decrease in the level of αKGM, the content of αKG in the liver of rats with HE was significantly (up to ~12 times) higher than the control values. In this regard, the ratios of αKG/αKGM metabolites in the liver tissues of the control group of rats (0.00115) and in the group of rats with chronic HE (0.01677) differed by ~14 times. As already noted, the development of HE has a stronger inhibitory effect on the activity of GTK due to its initially lower specific activity.

The specific activity of GTK in rat kidneys was one of the highest compared to other tissues; therefore, the predominance of specific activity of ωA over GTK in rat kidneys was not as significant as in the liver. In control samples of rat kidney tissue, this prevalence was ~6-fold, and in samples of rats with chronic HE, it was ~9-fold. As noted earlier, the activity of GTK was more sensitive to the effects of TAA than the activity of ωA. The concentration of αKGM in the kidneys of TAA-induced rats was reduced by ~2.5 times compared to the control group, while the concentration of αKG, on the contrary, was increased by about the same amount (~two times). Thus, the αKG/αKGM ratios between the control (0.8) and TAA-induced (3.4) groups of rats differed by almost ~4.5 times. For better visualization of changes in the αKG/αKGM (R) ratios in the blood plasma and organ tissues of control and TAA-induced rats, the following series is presented:

Plasma (R = 15.5) → Liver (R = 14.5) → Kidney (R = 4.5) → Brain (R = 2)

It is interesting to note that the R values in the blood plasma and liver samples of rats with chronic HE practically coincided. This suggests that blood plasma can be used to assess the severity of liver disease, which greatly simplifies the sampling procedure for analysis. It is planned to carry out testing on the blood plasma of patients with severe liver disease to confirm or refute the patterns identified in the model of TAA-induced rats. If the result obtained is confirmed, this ratio can not only be used for diagnostic purposes but also for monitoring the success of therapeutic interventions in various liver pathologies.

## 5. Conclusions

The development of chronic HE in TAA-induced rats was accompanied by a shift in the balance of the TCA cycle and associated metabolites. Conventionally, all considered metabolites can be subdivided into: (a) “process-dependent”, the change in the balance of which during the development of chronic HE had similar trends in all the considered samples; and (b) “organ-dependent”, the change in the balance of which depended on the location (organ) of the metabolite.

The first group of metabolites included Cit and αKG, αKGM, and Fum, the behavioral dynamics of which were similar in all types of samples studied: with the development of chronic HE, the levels of Cit and αKG increased relative to the control analogs, while the levels of αKGM and Fum decreased.

The second group of metabolites included Pyr, Lac, OA, and Form. The behavior of these metabolites during the development of chronic HE was determined by their localization. Thus, in the blood plasma and brain tissues of rats with chronic HE, the levels of Form increased against the background of a decrease in the levels of Pyr, Lac, and OA, and in the tissues of the liver and kidneys, the pattern of their distribution was mixed. In the liver, the levels of Pyr, OA, and Form increased with a decrease in the level of Lac, while in the kidneys, the distribution of these metabolites was reversed: the levels of Pyr, OA, and Form decreased with increasing levels of Lac.

For the αKG and αKGM metabolites included in the Gln-Glu cycle, as well as their ratios (αKG/αKGM), a certain bioindicative significance was revealed. Thus, under the conditions of the development of chronic HE in the liver, kidneys, brain, and blood plasma of rats, the level of αKG increased while the level of αKGM decreased.

The specific activity of the enzymes ωA and GTK, which regulate the levels of αKG and αKGM, was also significantly reduced in the tissues of the liver and kidneys but not in the blood plasma and brain of rats with chronic HE.

It has been suggested that the αKG/αKGM ratio may have an indicative value for assessing the degree of HE development. A remarkable feature of this ratio is that its values in blood plasma and liver tissues practically coincided. This circumstance allows, if necessary, to monitor the degree of development of HE in the blood plasma, avoiding the need to take tissue samples.

It is assumed that the patterns of distribution of αKG and αKGM identified in the model of rats with chronic HE may need an additional clinical study on blood plasma samples from humans with liver pathology.

## Figures and Tables

**Table 1:** AVG ± STD and confidence intervals at the 5% significance level for P(T < C).

P(T < C)	AVG ± STD *				Confidence Intervals *			
Metrics	Plasma	Liver	Kidney	Brain	Plasma	Liver	Kidney	Brain
Cit	0.28 ± 0.05	0.04 ± 0.02	0.38 ± 0.05	0.30 ± 0.04	(0.21, 0.37)	(0.01, 0.08)	(0.3, 0.47)	(0.22, 0.38)
αKGM	0.90 ± 0.03	0.60 ± 0.04	0.98 ± 0.02		(0.84, 0.95)	(0.52, 0.69)	(0.94, 1.0)	
Suc	0.61 ± 0.05				(0.52, 0.7)			
Fum		0.86 ± 0.03	0.61 ± 0.04	0.70 ± 0.04		(0.79, 0.91)	(0.53, 0.7)	(0.62, 0.78)
αKG	0.02 ± 0.02				(0.0, 0.06)			
αKG/αKGM	0.03 ± 0.03				(0.01, 0.08)			
Lac		0.75 ± 0.04	0.31 ± 0.04	0.67 ± 0.04		(0.66, 0.82)	(0.24, 0.4)	(0.59, 0.75)
Pyr	0.81 ± 0.04	0.13 ± 0.04	0.77 ± 0.04		(0.73, 0.87)	(0.08, 0.2)	(0.69, 0.84)	
Form	0.32 ± 0.04	0.40 ± 0.04		0.15 ± 0.04	(0.24, 0.4)	(0.32, 0.49)		(0.09, 0.22)
ωA		1.00 ± 0.02	0.93 ± 0.03			(0.97, 1.0)	(0.88, 0.97)	
GTK		1.00 ± 0.02	0.99 ± 0.02			(0.97, 1.0)	(0.96, 1.0)	

* Values are only presented in the cells where the hypothesis H_0_: P(T < C) = ½ was rejected with FDR control at the 5% significance level.

**Table 2:** The weights of the rats and their organs in the last weeks of the experiment.

Weight *	Rat	Liver	Kidney	Brain
14 weeks				
Control (*n* = 3)	310 ± 10	9.6 ± 0.3	1.7 ± 0.01	1.9 ± 0.1
TAA-induced (*n* = 7)	**178 ± 35**	10.5 ± 2.4	1.6 ± 0.2	1.8 ± 0.1
20 weeks				
Control (*n* = 3)	345 ± 21	10.4 ± 2.2	1.9 ± 0.2	1.1 ± 0.2
TAA-induced (*n* = 7)	**233 ± 18**	12.3 ± 2.2	1.8 ± 0.2	0.9 ± 0.1

* Weight in grams. Data are presented as the mean ± standard deviation (AVG ± STD). Those data are highlighted in bold, the AVG of which in the TAA group was statistically significantly different from the AVG of the control; explanation: |AVG_control − AVG_TAA| > 2 × (STD_control + STD_TAA), which corresponds to *p* < 0.05.

**Table 3:** Specific activity of ωA and GTK in the blood plasma, liver, kidney, and brain tissues of control and TAA-induced rats.

Enzyme/Sample	Plasma *	Liver **	Kidney **	Brain **
GTK				
Control	0.0029 ± 0.0014	0.0044 ± 0.0002	0.0118 ± 0.0015	0.0020 ± 0.0002
HE	0.0032 ± 0.0023	**0.0013 ± 0.0002**	**0.0046 ± 0.0013**	0.0018 ± 0.0002
ωA				
Control	0.0154 ± 0.0035	0.0672 ± 0.0048	0.0729 ± 0.0126	0.0045 ± 0.0015
HE	0.0145 ± 0.0066	**0.0241 ± 0.0060**	0.0433 ± 0.0123	0.0044 ± 0.0017

The specific activity of enzymes is expressed in nmols/mL/min * and nmols/mg of wet tissue/min **. Data are presented as the mean ± standard deviation (AVG ± STD). Those data are highlighted in bold, the AVG of which in the TAA group was statistically significantly different from the AVG of the control; explanation: |AVG_control − AVG_TAA| > 2 (STD_control + STD_TAA), which corresponds to *p* < 0.05.

**Table 4:** Total and mean concentrations of TCA cycle metabolites (*n* = 11) in blood plasma samples (μM *) and liver, kidney, and brain tissues (nmols per mg of wet tissue **) in groups of control and TAA-induced rats (11 cohorts).

Sample	Plasma *		Liver **		Kidney **		Brain **	
	Sum	Average	Sum	Average	Sum	Average	Sum	Average
Control	23,510.7	2137.3	57.7	8.3	59.9	6.0	156.7	17.4
HE	19,876.4	1806.9	65.9	7.3	63.9	6.4	145.6	16.2

**Table 5:** AVG ± STD data for deviation value ∆ = E(m((T − C)/C)).

E(m((T-C)/C))	AVG ± STD				Interpretation			
Measurement Type/Sample Type	Plasma	Liver	Kidney	Brain	Plasma	Liver	Kidney	Brain
Cit	***0.14 ± 0.11***	**13.07 ± 13.16**	0.25 ± 0.36	0.54 ± 0.81	***Mostly increased*** ***+14%***	**Strongly increased** **in 14 times**	*Increase stage dependent*	*Increase stage dependent*
αKGM	−0.49 ± 0.39	−0.10 ± 0.20	**−0.60 ± 0.10**		*Decrease stage dependent*	*Decrease stage dependent*	**Strongly decreased** **−60%**	
Suc	−0.07 ± 0.52				*Decrease stage dependent*	*No data*	*No data*	*No data*
Fum		**−0.38 ± 0.17**	0.02 ± 0.37	−0.10 ± 0.20	*Non-significant deviations*	**Strongly decreased** **−38%**	*Stage dependent*	*Decrease stage dependent*
αKG	**14.70 ± 10.65**				**Strongly increased** **in 16 times**	*No data*	*No data*	*No data*
αKG/αKGM	**43.64 ± 31.30**				**Strongly increased** **in 45 times**	*No data*	*No data*	*No data*
Lac		−0.12 ± 0.12	0.09 ± 0.12	−0.10 ± 0.15	*Non-significant deviations*	*Decrease stage dependent*	*Increase stage dependent*	*Decrease stage dependent*
Pyr	**−0.26 ± 0.13**	**10.21 ± 13.22**	−0.27 ± 0.26		**Strongly decreased** **−26%**	**Strongly increased** **in 11 times**	*Decrease stage dependent*	*Non-significant deviations*
Form	0.18 ± 0.23	0.15 ± 0.31		**0.19 ± 0.13**	*Increase stage dependent*	*Increase stage dependent*	*Non-significant deviations*	**Strongly increased** **+19%**
ωA		**−0.65 ± 0.10**	**−0.40 ± 0.13**		*Non-significant deviations*	**Strongly decreased** **−65%**	**Strongly decreased** **−40%**	*Non-significant deviations*
GTK		**−0.70 ± 0.06**	**−0.61 ± 0.10**		*Non-significant deviations*	**Strongly decreased** **−70%**	**Strongly decreased** **−60%**	*Non-significant deviations*
Cells terms explanation: *No data*—cells are marked for which there are not enough measurements for analysis. *Non-significant deviations*—cells for which P(T < C) = ½ are marked and have meaning that for a randomly selected pair of rats from the test and control groups from a randomly selected cohort, with equal probability, you can see a value in the test group lower than in the control and vice versa. *Decrease stage dependent* and, *Increase stage dependent*—cells in which P(T < C) ≠ ½ are indicated. Thus, in most cases, there is some deviation in the test measurements relative to the control. However, the changes in the remaining minority of cases do not allow the magnitude of the deviation to be recorded at or near the 5% statistical significance level. However, we can confidently talk about the sign of the deviation in most cases. **Bold font** indicates combinations of sample type and measurement type are highlighted in bold, where P(T < C) ≠ ½ and the cohort-average median deviation of measurements from the test group from the measurements of the control group is not equal to zero at the 5% significance level. On the right side of the Table 5 they are marked with the text Strongly increased/decreased. ***Bold italics*** indicate cells in which P(T < C) ≠ ½ and the cohort-average median deviation of measurements from the test group from the measurements of the control group is not equal to zero at a significance level of slightly more than 5% or greater tolerance for false positives; on the right side of the Table 5 they are marked with the text Mostly increased/decreased.								

## Data Availability

Data can be available upon personal request.

## Supplementary Materials

The following supporting information can be downloaded at: https://www.mdpi.com/article/10.3390/biom14020217/s1, Figure S1. Specific activity of ωA (A) and GTK (B) in brain tissues and blood plasma of control (black circle) and TAA-induced groups of rats (red triangle). The data are presented as average values (for the period of 2–22 weeks). Figure S2. Distribution of the pool of TCA metabolites in the blood plasma of control (black circle) and TAA-induced groups of rats (red triangle). The data are presented as average values (for the period of 2–22 weeks). Figure S3. Distribution of the pool of TCA metabolites in the liver of control (black circle) and TAA-induced groups of rats (red triangle). The data are presented as average values (for the period of 2–22 weeks). Figure S4. Distribution of the pool of TCA metabolites in the kidneys of control (black circle) and TAA-induced groups of rats (red triangle). The data are presented as average values (for the period of 2–22 weeks). Figure S5. Distribution of the pool of TCA metabolites in the brain of control (black circle) and TAA-induced groups of rats (red triangle). The data are presented as average values (for the period of 2–22 weeks).
