# Supplementary material for: Energy Metabolites and Indicative Significance of α-Ketoglutarate and α-Ketoglutaramate in Assessing the Progression of Chronic Hepatoencephalopathy
Source: Biomolecules. 2024 Feb 12;14(2):217. doi: 10.3390/biom14020217 (PMC10887089; doi:10.3390/biom14020217)
Supplement: Supplementary file 1 [file biomolecules-14-00217-s001.zip › biomolecules-2809691-supplementary.pdf]

Type of the Paper: Article

Energy Metabolites and Indicative Significance of α-Ketoglutarate and α-Ketoglutaramate in Assessing the Progression of Chronic Hepatoencephalopathy

Yevgeniya I. Shurubor [1,*], Andrey B. Krasnikov [2], Elena P. Isakova [3], Yulia I. Deryabina [3], Vladimir S. Yudin [1], Anton A. Keskinov [1], Boris F. Krasnikov [1,4,*]

[1] Centre for Strategic Planning of FMBA of Russia, Pogodinskaya St., Bld.10, 119121 Moscow, Russia; EShurubor@cspmz.ru (Y.I.S.); VYudin@cspfmba.ru (V.S.Y.); Keskinov@cspfmba.ru (A.A.K.); BKrasnikov@cspfmba.ru (B.F.K.)
[2] Independent Researcher, 24 Ivana Babushkina St., 117292 Moscow, Russia; kr-ab@yandex.ru (A.B.K.)
[3] Bach Institute of Biochemistry, Research Center of Biotechnology of the Russian Academy of Sciences, Moscow 119071, Russia; elen_iss@mail.ru (E.P.I.); yul_der@mail.ru (Y.I.D.)
[4] Department of Biochemistry and Molecular Biology, Faculty of Medicine, N.I. Pirogov Russian National Research Medical University, 1 Ostrovitianova Str., Moscow 117997, Russia; (B.F.K.)
* Correspondence: EShurubor@cspmz.ru (Y.I.S.) BKrasnikov@cspfmba.ru (B.F.K.)

SUPPLEMENTARY MATERIALS

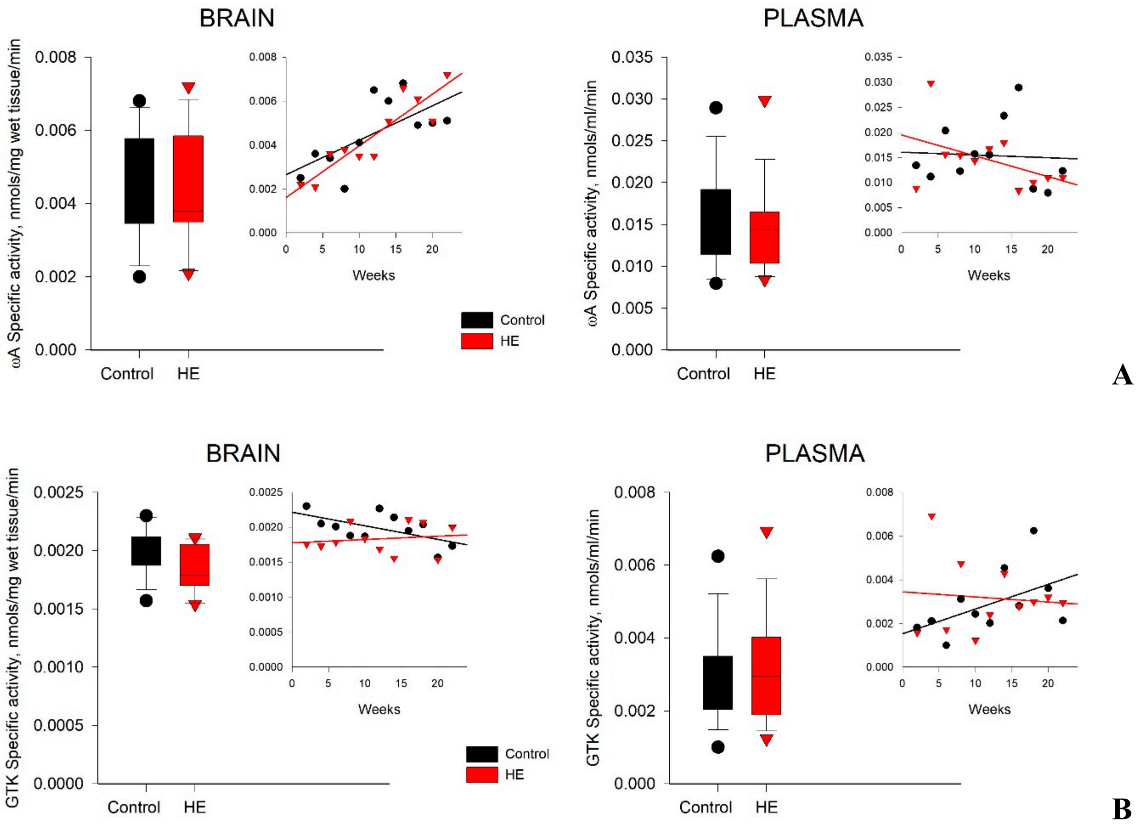

Figure S1. Specific activity of ωA (A) and GTK (B) in brain tissues and blood plasma of control (black circle) and TAA-induced groups of rats (red triangle). The data are presented as average values (for the period of 2-22 weeks).

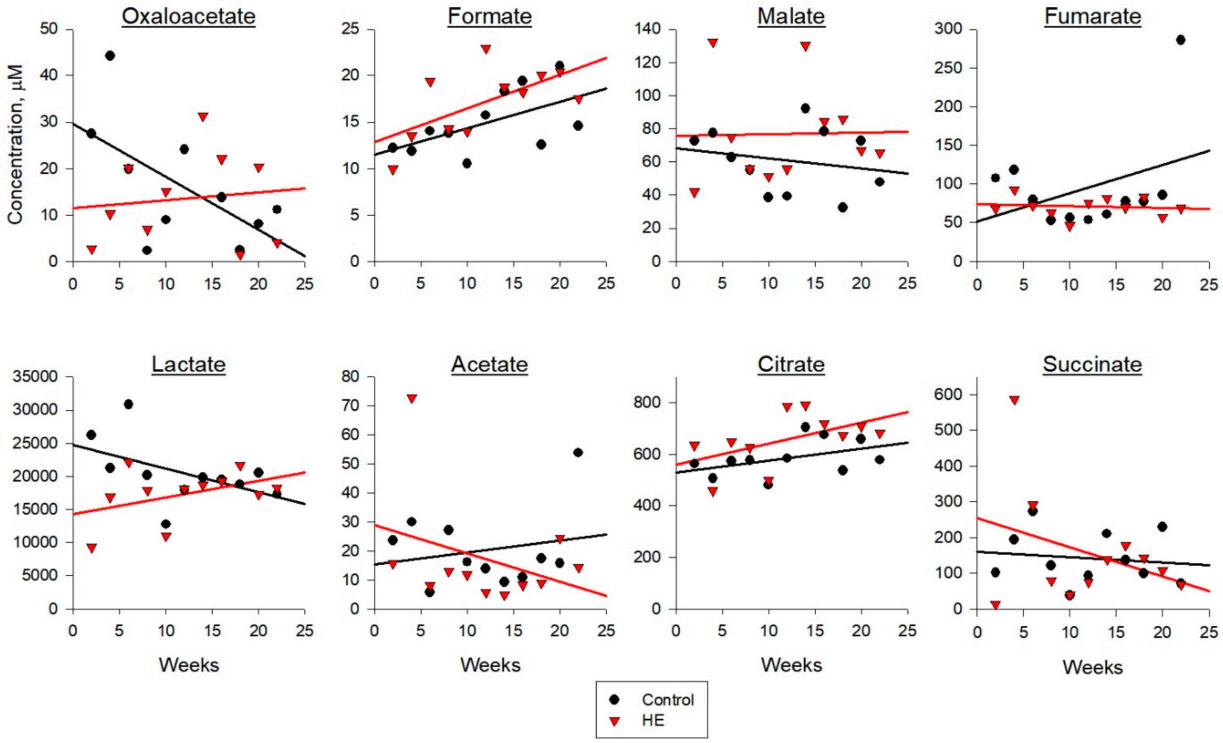

Figure S2. Distribution of the pool of TCA metabolites in the blood plasma of control (black circle) and TAA-induced groups of rats (red triangle). The data are presented as average values (for the period of 2-22 weeks).

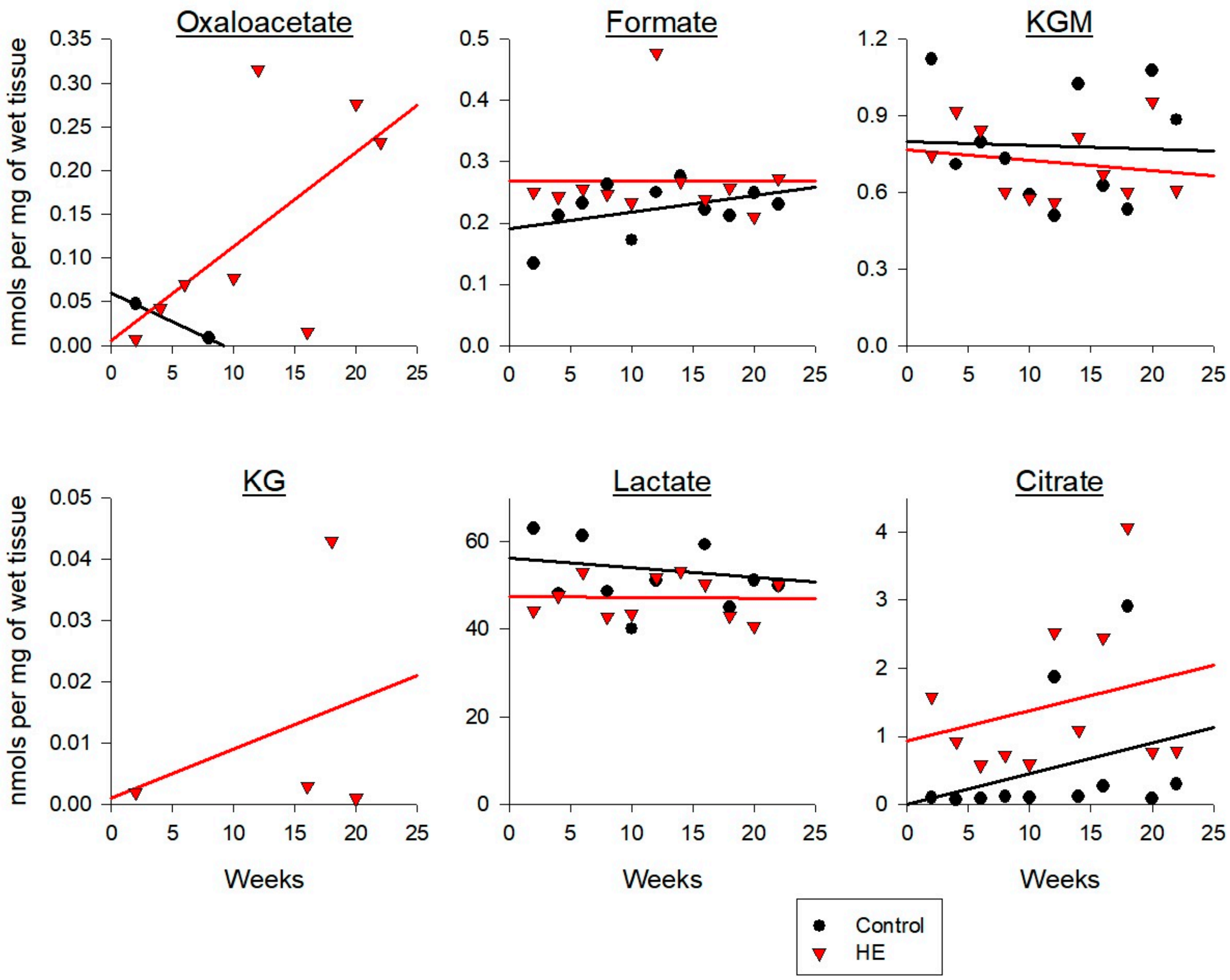

Figure S3. Distribution of the pool of TCA metabolites in the liver of control (black circle) and TAA-induced groups of rats (red triangle). The data are presented as average values (for the period of 2-22 weeks).

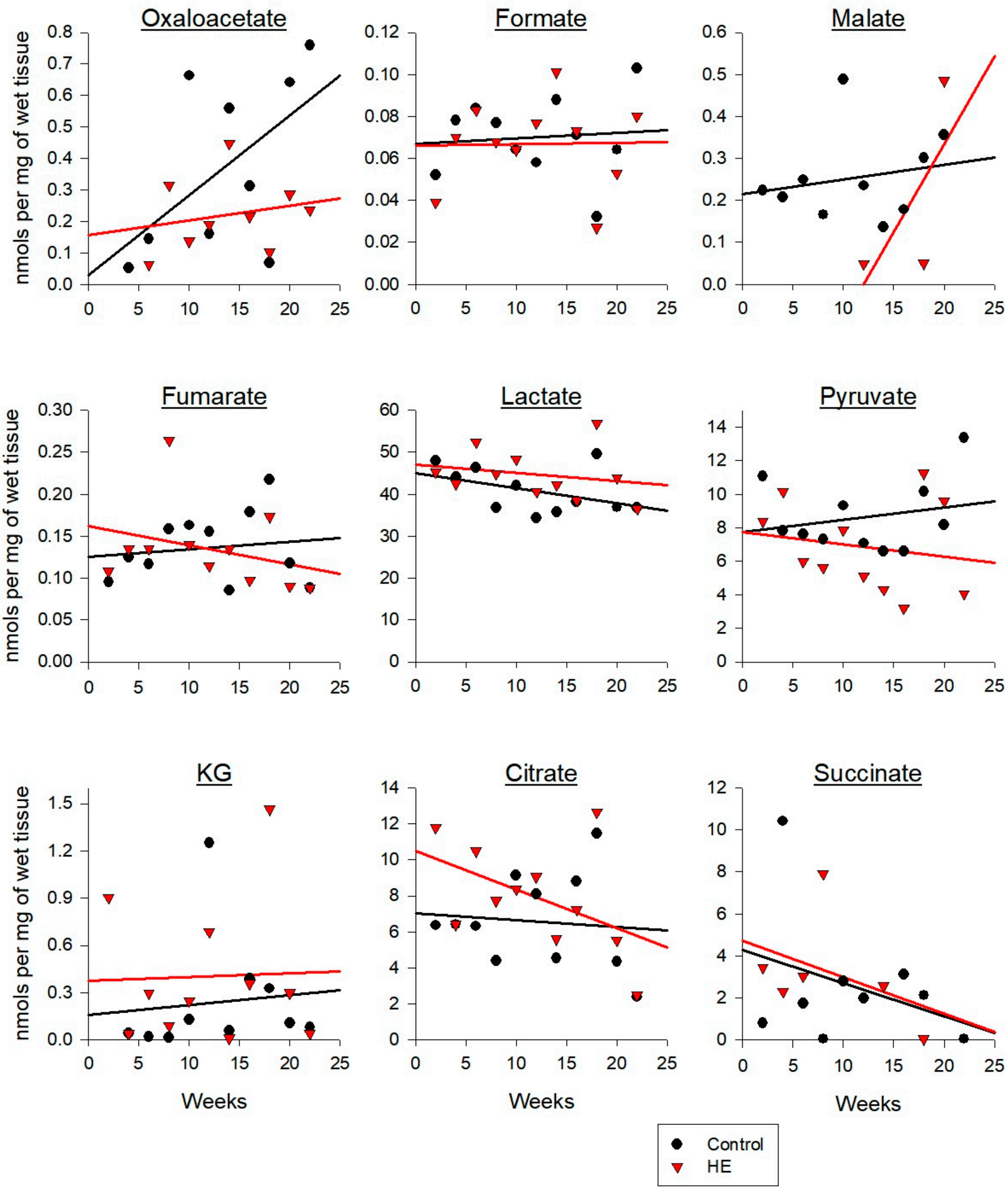

Figure S4. Distribution of the pool of TCA metabolites in the kidneys of control (black circle) and TAA-induced groups of rats (red triangle). The data are presented as average values (for the period of 2-22 weeks).

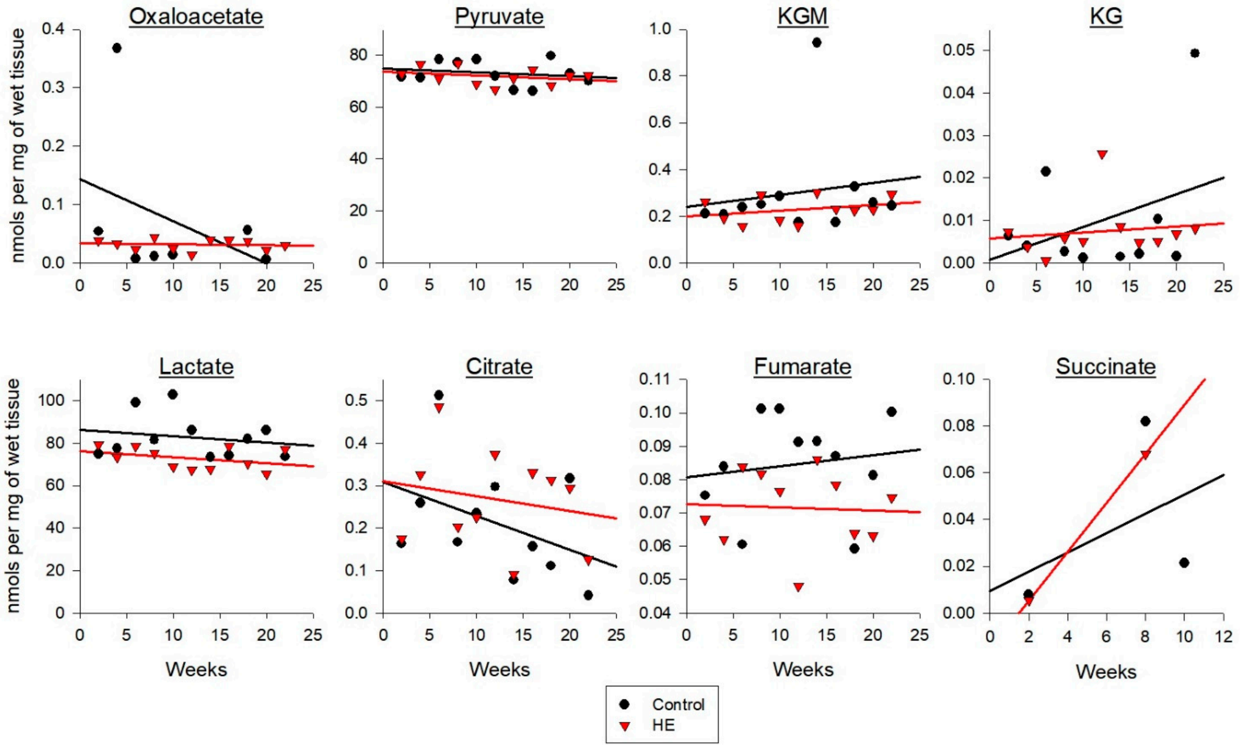

Figure S5. Distribution of the pool of TCA metabolites in the brain of control (black circle) and TAA-induced groups of rats (red triangle). The data are presented as average values (for the period of 2-22 weeks).
